# Calcium Dyshomeostasis in Tubular Aggregate Myopathy

**DOI:** 10.3390/ijms17111952

**Published:** 2016-11-22

**Authors:** Jong-Mok Lee, Satoru Noguchi

**Affiliations:** 1 Department of Genome Medicine Development, Medical Genome Center, National Center of Neurology and Neuropsychiatry, Kodaira, Tokyo 187-8551, Japan; azulmar@gmail.com; 2 Department of Neuromuscular Research, National Institute of Neuroscience, National Center of Neurology and Neuropsychiatry, Kodaira, Tokyo 187-8502, Japan

**Keywords:** tubular aggregate myopathy, skeletal muscle, severe combined immunodeficiency, STIM1, ORAI1, SOCE, calcium

## Abstract

Calcium is a crucial mediator of cell signaling in skeletal muscles for basic cellular functions and specific functions, including contraction, fiber-type differentiation and energy production. The sarcoplasmic reticulum (SR) is an organelle that provides a large supply of intracellular Ca^2+^ in myofibers. Upon excitation, it releases Ca^2+^ into the cytosol, inducing contraction of myofibrils. During relaxation, it takes up cytosolic Ca^2+^ to terminate the contraction. During exercise, Ca^2+^ is cycled between the cytosol and the SR through a system by which the Ca^2+^ pool in the SR is restored by uptake of extracellular Ca^2+^ via a specific channel on the plasma membrane. This channel is called the store-operated Ca^2+^ channel or the Ca^2+^ release-activated Ca^2+^ channel. It is activated by depletion of the Ca^2+^ store in the SR by coordination of two main molecules: stromal interaction molecule 1 (STIM1) and calcium release-activated calcium channel protein 1 (ORAI1). Recently, myopathies with a dominant mutation in these genes have been reported and the pathogenic mechanism of such diseases have been proposed. This review overviews the calcium signaling in skeletal muscles and role of store-operated Ca^2+^ entry in calcium homeostasis. Finally, we discuss the phenotypes and the pathomechanism of myopathies caused by mutations in the *STIM1* and *ORAI1* genes.

## 1. Introduction

The main roles of skeletal muscles are to produce the force for the maintenance of the body and for movement, in addition to many other functions. Calcium is a major signaling mediator of the force-generating contraction of skeletal muscles [1,2]. The Ca^2+^ released from the sarcoplasmic reticulum (SR) in response to depolarization of the transverse tubule (T-tubule) membrane by a nerve signal induces sarcomere contraction of myofibrils, a mechanism known as excitation–contraction (EC) coupling [3]. Ryanodine receptor 1 (RyR1) on the SR membrane releases Ca^2+^ from the SR lumen to the cytosol in skeletal muscles upon excitation, while the sarco/endoplasmic reticulum Ca^2+^ATPase (SERCA) transfers Ca^2+^ from the cytosol to the SR lumen by ATP hydrolysis during relaxation; thus, Ca^2+^ is recycled between the cytosol and the SR lumen. Upon depletion of the Ca^2+^ store in the SR, the cells take up extracellular Ca^2+^ to replenish the cytosolic stores via a mechanism called store-operated Ca^2+^ entry (SOCE) [4]. Several molecules essential for SOCE have been identified and characterized [5,6,7]. Recently, mutations in genes encoding two major proteins in SOCE, *stromal interaction molecule 1* (*STIM1*) and *calcium release-activated calcium channel protein 1* (*ORAI1*), have been identified as a cause of tubular aggregate myopathy (TAM) [8,9,10,11,12,13,14]. This review discusses the functions of STIM1 and ORAI1 in SOCE, the effects of their mutations on Ca^2+^ homeostasis in skeletal myofibers and the consequent pathomechanism of TAM.

## 2. Calcium Signaling in Skeletal Muscles

**Figure 1:**
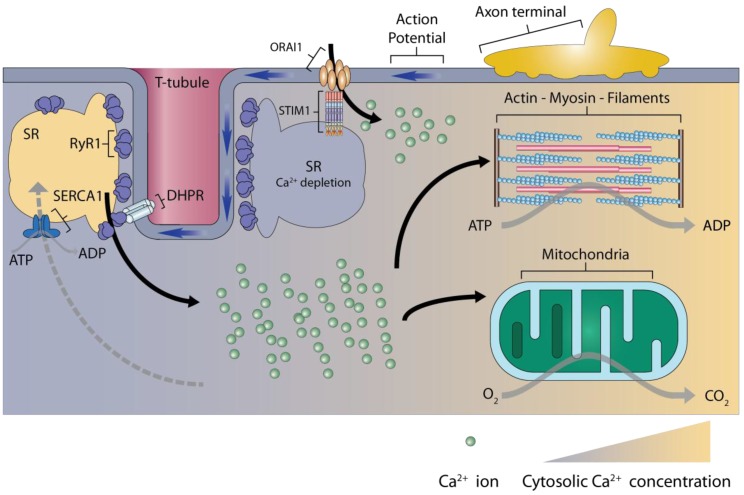
Diagram of excitation–contraction (EC) coupling in skeletal muscles. Depolarization of the action potential from the axon terminal induces the conformational change of the dihydropyridine receptor (DHPR) on transverse tubule (T-tubule) membranes. Calcium ions are released from the sarcoplasmic reticulum (SR) through ryanodine receptor 1 (RyR1), which is activated by DHPR binding. Released Ca^2+^ directly induces contraction. After EC coupling, released Ca^2+^ reuptake is transferred to the SR by sarco/endoplasmic reticulum Ca^2+^ATPase (SERCA1). Upon SR Ca^2+^ depletion, store-operated Ca^2+^ entry is mediated by conformation changes in stromal interaction molecule 1 (STIM1) that are communicated with calcium release-activated calcium channel protein 1 (ORAI1). ATP, adenosine triphosphate; ADP, adenosine diphosphate.

The contraction of skeletal muscles is mediated by a mechanism termed “EC coupling” in which action potential induced by the nervous system is turned into molecular interactions of myofilaments to generate a physical force. During EC coupling, the evoked potential runs along the sarcolemma and triggers the structural change of the dihydropyridine receptors (DHPR) or l-type Ca^2+^ channels, located in the T-tubule membrane. Then, the activation of DHPR mediates a physical interaction with RyR1 channels on the SR membrane, resulting in the opening of the RyR1 channels (Figure 1) [1]. The RyR1 channel opening enables Ca^2+^ release from the SR, resulting in a 30-fold increase in cytosolic Ca^2+^ levels compared to the normal range (i.e., from around 50–200 nM) [2]. The RyR1 density in type II fibers is much higher than that in type I fibers [3,4] and it has been considered that the Ca^2+^ release from RyR1 is associated with the fiber type differentiation.

Released Ca^2+^ from the SR via RyR1 channels has versatile functions and is necessary for force generation primarily by interactions between myosin and actin filaments. Upon Ca^2+^ release from the SR, two Ca^2+^ molecules bind troponin C, pulling the troponin–tropomyosin complex aside to allow myosin heads to bind to actin filaments (Figure 1) [5]. Binding of the actin and myosin cross-bridge triggers a power stroke that pulls thin filaments inward during contraction; splitting of ATP by myosin ATPase provides energy for the power stroke of the cross-bridge. This cross-bridge binding is maintained until a new ATP binds to myosin and replaces the ADP that is bound to the myosin heavy chain heads and the cycle can be repeated [6]. The repeated rate depends on the amount of Ca^2+^ that is bound to troponin C [5]. Therefore, the cross-bridge binding is facilitated by the controlled abundance of Ca^2+^, and the force generation in skeletal muscles depends on the Ca^2+^ concentration [7].

Following EC coupling, released Ca^2+^ from the SR has to be removed after the discontinuation of action potentials for the relaxation of sarcomeres and preparation of the next contraction [8]. Upon relaxation of the skeletal muscle, return of the cytosolic Ca^2+^ concentration to normal level allows tropomyosin to move back to its original position, blocking myosin cross-bridge binding sites on actin and allowing the thin filaments to passively slide back to their original position [6]. Ca^2+^ uptake across a 10,000-fold calcium gradient toward the SR is mainly controlled by SERCA1 in skeletal muscles [9]. SERCA1a, which is mainly expressed in fast twitch (type II) fibers, enables Ca^2+^ reuptake to be seven-fold higher in type II than in type I fibers, contributing to SR being more developed in type II fibers [10,11,12].

Dysfunction of the EC coupling system is the cause of several skeletal muscle disorders, including malignant hyperthermia (MH), central core disease (CCD), and Brody disease [13]. MH is a pharmacogenetic disorder, triggered by halogenated inhalational anesthetics or depolarizing muscle relaxants in predisposed individuals [13,14,15]. The symptoms consist of enhanced muscle metabolism, muscle rigidity, and dramatically increasing body temperature by 1 °C per 5 min [16]. The exposure to triggering agents initiates an uncontrolled increase in the cytosolic Ca^2+^ concentration that leads to increased muscle contraction and heat production associated with metabolic acidosis, excessive ATP hydrolysis and hypoxia [17]. If this disorder is not treated with the proper medication, dantrolene, an inhibitor of Ca^2+^ release from the SR, up to 70% of patients die [17]. Over 200 mutations in *RyR1* have been found in this disorder [18,19]. MH mutations are frequently located in the cytosolic region of the RyR1 channel and have a gain-of-function effect. However, the mutations in the C-terminus or SR luminal region of RyR1 cause CCD, characterized by hypotonia, proximal muscle weakness, skeletal abnormalities and delayed motor milestones [20]. Several mutations causing CCD are overlapped with the pathogenic mutations for MH [18,19,21]. CCD can be diagnosed by the pathological core hallmark, which shows an enzymatic deficiency in the NADH-tetrazolium reductase (NADH-TR) stain [22]. CCD patients lose the ability to release Ca^2+^ from the SR, leading to EC uncoupling [23,24]. Brody disease is a rare autosomal recessive muscle disorder characterized by painless contractures and impairment of relaxation during exercise [25]. Later, point or nonsense mutations in *ATPase sarco/endoplasmic reticulum Ca^2+^ transporting 1* (*ATP2A1*) have been identified in patients with Brody disease, resulting in the failure of SERCA activity to keep up with the Ca^2+^ release during repetitive stimulation [26,27].

Calcium ion uptake by mitochondria has a minor role in the clearance of cytosolic Ca^2+^ during EC coupling [27]. Nevertheless, this uptake is sufficient and necessary for the maintenance of energy homeostasis in contracting skeletal muscles [28]. Raised Ca^2+^ levels in the mitochondrial matrix activates the mitochondrial dehydrogenase, leading to accelerated nicotinamide adenine dinucleotide reduction and oxidative phosphorylation [29,30]. Additionally, increased mitochondrial matrix Ca^2+^ stimulates ATP synthesis through the activation of F1F0-ATP synthase [31]. Although the mechanism in the expanded notion of “excitation–contraction–metabolism coupling” remains unknown, Ca^2+^ is obviously involved in the regulation of the mitochondrial respiratory chain [2,27,29].

## 3. Store-Operated Calcium Entry

### 3.1. The Main Components of Store-Operated Calcium Entry

**Figure 2:**
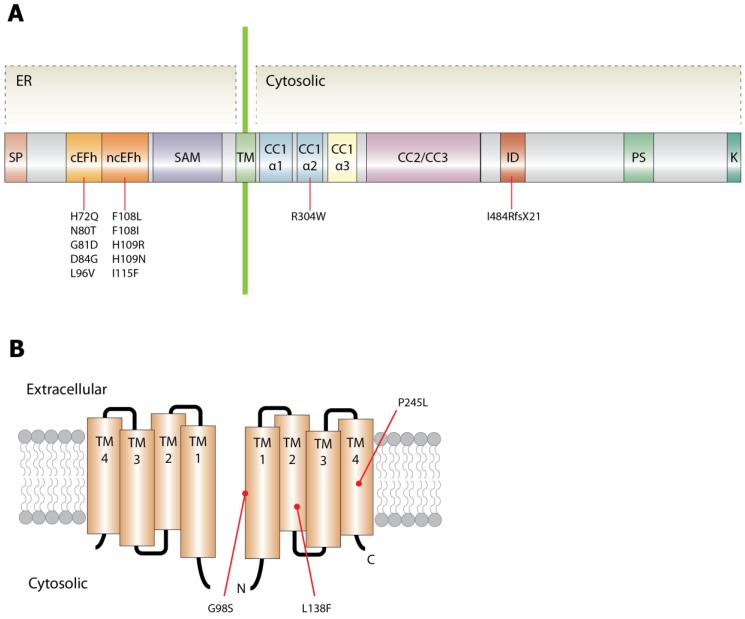
Schematic structures of STIM1 and ORAI1. A single straight STIM1 molecule is depicted. The major site for binding Ca^2+^ is a canonical EF-hand domain (**A**). Two ORAI1 subunits out of six are illustrated. ORAI1 protein has four transmembrane helices with the N- and C-termini located in the cytoplasm (**B**). The locations of reported mutations are marked (**A**,**B**). SP, signal peptide; cEFh, canonical EF-hand; ncEFh, noncanonical EF-hand; SAM, sterile α-motif; TM, transmembrane; CC, coiled-coil region; ID, inhibitory domain; PS, Pro/Ser-rich domain; K, Lys-rich domain.

Maintenance of adequate cytosolic Ca^2+^ is necessary for sustained contractility of skeletal muscle fibers. In addition to the fast activation of EC coupling rendered by DHPR and RyR1s, SOCE has an important role in maintaining contractile function by facilitating Ca^2+^ influx from the extracellular area during SR Ca^2+^ depletion, although the SOCE has slower kinetics [32,33,34,35]. SOCE is primarily facilitated by the interactions among STIM1, ORAI1, and canonical transient receptor potential channels (TRPC) in skeletal muscles [36,37,38,39]. STIM1 is a single transmembrane (TM) protein consisting of 685 amino acids (NP_003147.2) and localized in the sarco/endoplasmic reticulum (SR/ER) (Figure 2A). The N-terminus of this protein includes a Ca^2+^ sensing domain facing the SR/ER luminal side, forming canonical EF-hand (cEFh), non-canonical EF-hand (ncEFh), and sterile α-motif (SAM) domains. The C-terminus in the cytoplasm provides the binding sites for other cytosolic proteins [32,33,34,35,40]. ORAI1 has a tetra-spanning transmembrane (TM1–TM4) structure, which consists of 301 amino acids (NP_116179.2, Figure 2B), and forms Ca^2+^-selective ion channels as multimers in the plasma membrane [41,42]. Each transmembrane domain is connected by one intracellular and two extracellular loops, attaching the cytosolic N- and C-termini. The crystal structure of *Drosophila* ORAI1 suggests that the ion channel is composed of six homophilic molecules [43,44,45,46]. Its central ion pore is made of a ring consisting of six TM1 domains, surrounded by a second ring formed by six TM2 and TM3 domains, which is encircled by an outer TM4 ring [44,47,48]. The C-terminus in the cytoplasm connects to TM4 via a conserved hinge region, binding to the C-terminus of an adjacent monomer [40]. Gly98, located in TM1, has been well characterized as a gating hinge for the channel [49]. Replacing this residue with alanine results in channel activity failure, while aspartate or proline replacements exhibit a negative charge or hydrophilic property, causing constitutive channel opening and reduced ion selectivity in a STIM1-independent manner [43,45,47,50,51,52].

### 3.2. Activation of Store-Operated Calcium Entry by STIM1 Binding to ORAI1

Upon Ca^2+^ depletion in the SR, the SOCE pathway is activated to replenish the cytosolic Ca^2+^ stores, which is important during prolonged tetanic stimulation and fatigue [43,52,53,54,55,56,57]. The luminal cEFh domain of STIM1 loses the Ca^2+^ binding and undergoes conformational changes to the unfolded form, exposing hydrophobic residues [58,59]. This triggers aggregation of the luminal STIM1 domains, which is the first step in the SOCE activation cascade [58,59,60]. The coiled-coil (CC) CC2/CC3 domain has a major role in mediating this oligomerization [61,62]. The result obtained using serial C-terminus truncated STIM1 mutants disclosed that the CC1 domain alone cannot oligomerize [62,63]. In this conformational change, the inhibitory clamp in the STIM1 C-terminus is released from the tight and inactive state to the extended and active state. Luminescence resonance energy transfer (LRET) studies using CC2/CC3 domain (aa 353–450) fragments labeled at both ends have revealed a reduction in the intermolecular LRET upon binding to ORAI1 or substitution of specific residues (Lys251), supporting this mechanism [60,64]. Expression of the CC2/CC3 domain of STIM1, which is also called as the ORAI1 activating small fragment (OASF), Ca^2+^ release-activated Ca^2+^ (CRAC) activation domain (CAD), the STIM1-ORAI1 activating region (SOAR) or the CC domain b9 (CCb9), has been reported to be sufficient for constitutive ORAI1 channel activation [64,65,66]. In contrast, both the N- and C-terminus of ORAI1 are essential for the channel activity as they contain functional binding sites for activated STIM1 [40,67]. Carboxy-terminally deleted ORAI1 could not interact with STIM1 [40,64,68]. In contrast, an N-terminal deletion mutant was still capable of interacting with STIM1, forming clusters [40]. These studies suggest that the C-terminus of ORAI1 has stronger binding sites to STIM1 than the N-terminus. However, neither N- nor C-terminal deletion mutants of ORAI1 generated a Ca^2+^ influx. The interaction between STIM1 and TRPCs contributes to SOCE [69,70]. Moreover, interaction among TRPC, STIM1, and ORAI1 is associated with the SOCE [32]. The interplay of ORAI1 with TRPC3 and TRPC6 has also been reported [36]. The precise mechanism of these interactions remains unknown.

Autosomal recessive mutations in *STIM1* or *ORAI1* result in impaired or abolished SOCE in immune cells and non-immune cells. Affected individuals with loss-of-function mutations in these genes are known to have severe combined immunodeficiency (SCID), characterized by a defect in T-cell activation, recurrent infections [43,45,46], ectodermal dysplasia [47,48], abnormal enamel [43,45,47,52], and muscle hypotonia [43,52,54,56,57]. Transgenic mice with muscle-specific expression of dominant negative ORAI1 have been reported to exhibit reduced body weight, muscle mass, and fiber cross-sectional area [58]. These mice displayed increased susceptibility to fatigue during repetitive activity, which may have been due to SR Ca^2+^ depletion [58].

## 4. Tubular Aggregate Myopathy

### 4.1. Diagnosis and Pathological Features

Currently, the diagnosis of TAM, itself, is accomplished by identifying tubular aggregates (TAs) in a muscle biopsy; TAM can be suspected in patients with typical phenotypes, such as periodic paralysis, congenital myotonia, or malignant hyperthermia [71,72]. However, this diagnosis should be considered in patients presenting proximal dominant leg weakness, which may be confused with other types of muscular dystrophy or inflammatory myopathy in most cases of *STIM1* or *ORAI1* mutations.

**Figure 3:**
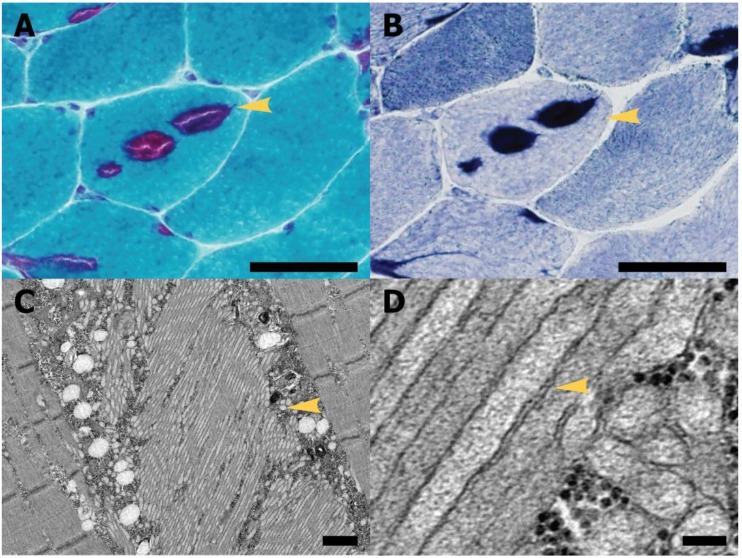
Histology and electron microscopy of a muscle biopsy from a patient with tubular aggregate myopathy. Histological analysis reveals tubular aggregates (TAs) in modified Gomori trichrome (**A**) and NADH-tetrazolium reductase (NADH-TR) (**B**) stainings; using electron microscopy, TAs are demonstrated as a cluster of single-walled tubules in parallel direction (**C**), which is also shown in high magnification (**D**). Arrowheads indicate TAs. Scale bars, 50 µm (**A** and **B**), 1 µm (**C**), and 0.1 µm (**D**).

A distinctive pathological feature of TAM is the presence of TAs in myofibrils [73]. Under a light microscope, TAs are recognized as granular or inclusions of bright red materials located centrally or peripherally in modified Gomori trichrome staining, and as an intense enzymatic reaction in NADH-TR staining (Figure 3A,B). Using electron microscopy, TAs are observed as straight single- or double-walled tubules aligned in parallel in a longitudinal section and a honeycomb-like structure in the transverse dimension (Figure 3C,D). In addition to TA formation, TAM shows increased variation in fiber size, regenerating fibers, increased number of fibers with internalized nuclei, endomysial fibrosis, and type 1 fiber predominance, indicating that TAM is a progressive disease. STIM1 and ORAI1 are colocalized with SERCA or DHPR in immunofluorescent staining [61], suggesting that TAs originate from disruption of the sarcotubular system owing to altered Ca^2+^ homeostasis [74,75], although the precise mechanism of TA formation is unclear. TAs have often been found in genetically heterogeneous neuromuscular disorders of an autosomal-dominant inheritance, and are well explained in neuromuscular disease associated with gain-of-function *STIM1* or *ORAI1* mutations as aspects of Ca^2+^ dyshomeostasis.

### 4.2. Clinical Manifestations

The clinical spectrum of TAM with mutations in *STIM1* varies from asymptomatic to slowly progressive limb weakness or Stormorken syndrome. The most common presentation (50%, 12/24) is weakness of the proximal dominant lower limbs mimicking other types of muscular dystrophy [76,77,78,79,80]. Even in this group, the onset age varies from childhood to adulthood, and the severity of muscle weakness ranges from mild weakness to inability of self-ambulation [77,79,81]. The second presentation is extra ocular muscle (EOM) weakness, which accompanies limb weakness (7/24). Upward or lateral gaze palsy, ophthalmoplegia, and ptosis have also been revealed. Walter et al. have reported two members in one family with the same mutation, showing different involvement of the ocular muscle: the father showed a severe proximal limb weakness with EOM palsy, whereas the son did not complain of gaze paresis [80]. Also, patients from a different family harboring the same *STIM1* Asn80Thr mutation showed different involvement of EOM [77]. Interestingly, most patients suffering from EOM weakness show multiple joint contracture and more severe limb weakness in comparison with those with limb muscle weakness only. EOM involvement appears to depend not on the mutation location, but rather on the weakness duration. The third presentation (5/24) is normal muscle strength with or without myalgia or fatigue. One patient with *STIM1* His109Asn had not shown muscle weakness, but myalgia, fatigability, or episodic diplopia [77]. One family with *STIM1* His72Gln showed elevated serum creatine kinase (CK) levels without muscle weakness [76]. An affected individual with *STIM1* Phe108Leu had shown myalgia without muscle-related symptoms, including weakness or elevated serum CK [77]. An imaging study using muscle magnetic resonance imaging and computerized tomography, which are useful for clarifying presymptomatic involvements, identified fatty replacement of obturator, gluteus, sartorius, gastrocnemius, soleus, and peroneus in early stages in patients with mutations in STIM1 luminal domains [77,78]. In advanced stages, low involvement of thigh muscles and tibialis posterior was found, whereas adductor longus, gracilis, short head of the biceps femoris, tibialis anterior, extensor halluces, and digitorum longus muscles were relatively spared. Interestingly, the involvement of flexor hallucis longus was found in patients with *STIM1* mutations, but not in non-*STIM1* patients. Upper girdle imaging has revealed that the subscapularis was affected, while masticator and trapezius were spared in patients with *STIM1* mutations [82]. In laboratory results, the serum CK level was elevated from 4- to 15-fold of the upper normal limit in most patients with *STIM1* mutations [76,77,79,80].

Stormorken syndrome, which symptoms comprise small stature, bleeding tendency, muscle fatigue, mitosis without ptosis, asplenia, headache, dyslexia, and ichthyosis, is the most severe form of the disease spectrum seen with *STIM1* mutations [83]. Thrombocytopenia or thrombocytopathia is frequently observed in most patients with a bleeding tendency [64,84,85]. So far, patients with the *STIM1* Arg304Trp mutation reported by Misceo et al. have shown hematoma after minor trauma, recurrent epistaxis, subarachnoid hemorrhage, and skin purpura [84]. One patient underwent a splenectomy to treat purpura before the Stormorken syndrome was identified. Another patient harboring the same mutation described by Nesin et al. also exhibited a bleeding diathesis [64].

The clinical features of patients with *ORAI1* Gls98Ser and Leu138Phe mutations showed slowly progressive diffuse muscle weakness [61]. Computerized tomography imaging of a patient with an *ORAI1* mutation showed severe atrophy and fat infiltration into the gluteus medius, hamstrings, and adductor muscles in the thigh. Mild serum hypocalcemia was present in all patients and may be associated with decreased parathyroid hormone secretion, although the precise pathomechanism is unclear. Other symptoms in patients with the *ORAI1* Gly98Ser mutation include calcification in the brain and intellectual disability related with Stormorken syndrome, but no bleeding tendency or miosis [61]. Patients with the *ORAI1* Pro245Leu mutation showed miosis without bleeding diathesis, indicating a Stormorken-like syndrome, which had been reported previously without genetic analysis [64,86]. Six members were affected with an autosomal dominant inheritance pattern and showed a small pupil size with a 1 mm bilateral resistance to the pupil dilators tropicamide and phenylephrine [86]. Cardiac or respiratory involvement is not common among patients with *STIM1* or *ORAI1* mutations.

## 5. Genetic Causes and Possible Molecular Mechanism of Tubular Aggregate Myopathy

### 5.1. STIM1

**Figure 4:**
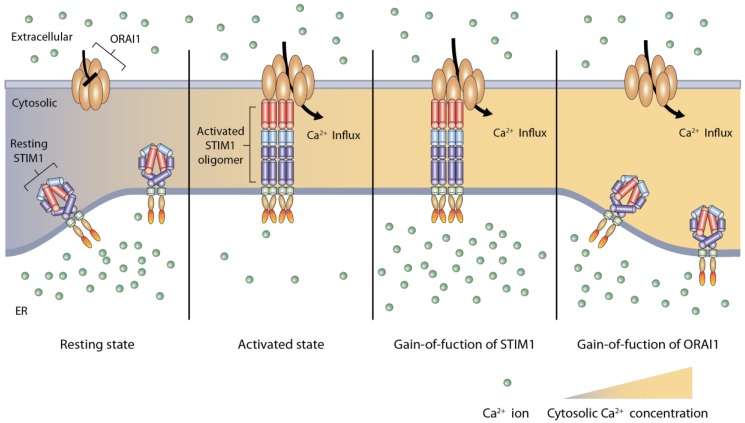
Schematic representation of Ca^2+^ overload in tubular aggregate myopathy. STIM1 molecules are in a tight and stable conformation in the resting state when there is an abundance of sarcoplasmic reticulum (SR) Ca^2+^. Upon depletion of SR Ca^2+^, STIM1 is activated and undergoes conformational changes to an elongated shape, binding with ORAI1 in the activated state. Mutated STIM1 can be activated without SR Ca^2+^ depletion, resulting in ORAI1 channel opening (gain-of-function of STIM1). Gain-of-function caused by mutations in ORAI1 allows extracellular Ca^2+^ influx to the cytosol, which is independent of the SR Ca^2+^ concentration or STIM1 activation (gain-of-function of ORAI1).

Most of the autosomal dominant mutations reported in TAM are located in the EF-hand domain of STIM1, which is considered to be a hotspot. So far, ten different mutations have been reported. Five (His72Glu, Asn80Thr, Gly81Asp, Asp84Gly and Leu96Val; Figure 2A) are positioned in the cEFh and only one of them affects a negatively-charged residue (Asp84) that coordinates Ca^2+^ binding [76,77,80,84]. The other five mutations (Phe108Ile, Phe108Leu, His109Asn, His109Arg and Ile115Phe; Figure 2A) are located in the ncEFh, which contributes to the stability of the cEFh domain. Overexpression of yellow fluorescent protein-tagged STIM1 proteins with the His72Glu, Asn80Thr, Asp84Gly, Leu96Val, Phe108Ile, His109Asn, or His109Arg mutation has resulted in clustering without SR/ER Ca^2+^ depletion [64,76,77]. C2C12 myoblasts transfected with the Asp84Gly mutant have shown elevated cytosolic Ca^2+^ levels, supporting activation of CRAC channels (Figure 4) [61,76]. A mouse model harboring the same Asp84Gly mutation has revealed thrombocytopenia and a bleeding disorder, but the skeletal muscles have not been investigated [64,87]. In myoblasts from an affected individual harboring the Asp84Gly mutation, functional characterization by calcium imaging has revealed a constitutive Ca^2+^ influx and marked increases in cytosolic Ca^2+^ upon treatment with thapsigargin, a SERCA inhibitor (Figure 4) [80,86]. In contrast, a heterozygous mutation (Ile484ArgfsX21; Figure 2A) located in the C-terminal inhibitory domain has been reported in TAM and HEK239 cells expressing this *STIM1* mutant have shown decreased intracellular Ca^2+^ influx, although the precise mechanism needs to be investigated [79,88]. Therefore, *STIM1* mutation may also cause TAM in spite of the down-regulation of the cytosolic Ca^2+^ level.

A cytosolic mutation (Arg304Trp; Figure 2A) has been reported to result in Stormorken syndrome. This mutation is located in the CC1 domain and results in constitutive Ca^2+^ influx without SR/ER Ca^2+^ depletion [64,84,85,89]. Store-operated Ca^2+^ entry induced by thapsigargin treatment was moderately increased in HEK293 cells transfected with this mutant [64,85,90]. These cells have shown the presence of STIM1 puncta in both thapsigargin-treated and untreated cells [65,66,85,91]. Thus, these data indicate that the Arg304Trp mutation results in constitutive Ca^2+^ influx. The pathomechanism of the Arg304Trp mutation can be ratiocinated based on a clamp structural model. Arg304 in the CC1 domain contributes to stabilizing the STIM1 protein in a tight conformation. This residue creates a salt bridge with Glu318 and makes a hydrogen bond with Glu314. Tryptophan replacement of this residue can hinder both of these interactions, changing the conformation of the cytosolic domain to be elongated and unstable. As discussed above, Leu251Ser in STIM1 also results in constitutive Ca^2+^ influx by a conformational change from a stable and tight clamp to an unstable and elongated conformation of the cytosolic domain [60].

Additionally, an experiment on the bleeding tendency in Stormorken syndrome has been performed to explain the dysfunction of platelets. The platelets from patients have been found to be in a procoagulant state without stimulation mediated by exposure to phosphatidylserine [84,92]. Glycoprotein 53 and P-selectin, which are platelet activation markers, were elevated and α-granule secretion was found in unstimulated patients’ platelets [84]. The dysfunction of platelets with the Arg304Trp mutation is summarized in an animal model. Nesin et al. have generated zebrafish embryos by injecting STIM1 Arg304Trp mRNA. The affected zebrafish embryos revealed brain or caudal hemorrhage, and a reduction in thrombocyte numbers, which is consistent with the phenotype of Stormorken syndrome [64]. Of note, heterozygous Ile115Phe and Arg304Trp mutations have been reported to be shared by York platelet syndrome, characterized by thrombocytopenia with ultrastructural platelet abnormalities, including giant electron-opaque organelles, multilayered target bodies, and poor Ca^2+^ storage in delta-granules [93]. Elevated expression of platelet markers in glycoprotein 53 and P-selectin obtained from the York platelet syndrome was overlapped with the experimental results of Stormorken syndrome [84]. The myopathic symptoms in the York platelet syndrome are clinically similar to those in TAM, consisting of proximal dominant limb weakness, recurrent falls, ophthalmoplegia, and miosis, although the pathologic findings do not reveal typical TAs but the presence of rimmed vacuoles. [93]. These findings may expand the phenotypic spectrum of STIM1-related disorders.

### 5.2. ORAI1

Three different autosomal dominant mutations in *ORAI1* in four different families have been reported to cause the TAM phenotype [61,64]. The Gly98Ser and Leu138Phe mutations resulted in constitutive external Ca^2+^ influx without SR/ER Ca^2+^ depletion in the patients’ myotubes and in HEK293 cells transfected with these mutant proteins [61]. This Ca^2+^ influx can be suppressed with CRAC blockers, indicating that the Ca^2+^ influx is mediated by CRAC channels. The Gly98Ser mutation in TM1 may give a hydrophilic property to the channel pore, similar to a proline replacement (Figure 2B). The second mutation, Leu138Phe, is located in TM2 (Figure 2B). The mechanism of CRAC channel opening by this mutation is unclear and remains to be elucidated. Additionally, Trp176 and Gly183 in TM3 have been reported to alter the properties of the CRAC channel. The Trp176Cys mutant has exhibited Ca^2+^ influx in the absence of STIM1, whereas the Gly183Ala mutant has exhibited Ca^2+^ influx in the presence of the CRAC channel blocker, 2-aminoethoxydiphenyl borate [63]. These results indicate that mutations of residues in TM3 can affect the properties of the channel, although the residues in TM3 are not components of the CRAC channel pore.

The third *ORAI1* mutation in TAM is located in TM4 (Figure 2B) [64]. HEK293 cells transfected with the Pro245Leu mutant did not result in constitutive Ca^2+^ influx, which was observed in other mutations. This mutation induced an enhanced and prolonged SOCE after passive SR/ER store depletion with thapsigargin. The altered inactivation of the mutant CRAC channel may contribute to the prolonged elevated cytosolic Ca^2+^. The slow Ca^2+^-dependent inactivation (CDI) was also significantly reduced in HEK293 cells transfected with the Pro245Leu mutant, whereas the fast CDI was normal. These findings indicate that prolonged opening of CRAC channels by this mutation causes increased intracellular Ca^2+^ concentrations [64]. The mechanism by which the mutations of residues in TM4 affect the channel opening and closing has been suggested: Pro245 stabilizes the TM4 ⍺-helix in a kinking shape and its shape restricts the movement of TM2 and 3. This change locks TM1 in place with the side chain of Val102 projecting towards the pore to prevent ion permeation. The opening of ORAI1 is induced by binding of CAD to the N-terminal and C-terminal of ORAI1 causing conformational changes in TM4 from kinking to straight. The hydrophobic side chain of Val102 is repositioned away from the pore, allowing ion permeation [67]. The Pro245Leu mutation cannot maintain TM4 at the kinking state and, thus, allows Ca^2+^ influx.

What turns the phenotypes into TAM, alone, or Stormorken syndrome remains unclear. An explanation has been elucidated from the difference in the fast CDI between the STIM1 CC1 mutation of Arg304Trp and the EF-hand mutation of Asp76Ala. STIM1 Asp76Ala showed a fast CDI similar to wild type STIM1, whereas Arg304Trp mediated a reduced fast CDI. These data suggest that prolonged opening of the CRAC channel in the STIM1 Arg304Trp mutant mediates more severe cytosolic Ca^2+^ overload than the STIM1 EF-hand mutant or the wild type; therefore a broad phenotype spectrum in Stormorken syndrome is seen [64].

The progression of TAM and the contribution of genetic factors to its variability need to be further delineated.

## 6. Closing Remarks

Ca^2+^ is essential for skeletal muscles. Multiple roles in the regulation of contraction, metabolism, and plasticity [94] are coordinated and well explained by the interplay between Ca^2+^ release from the SR and SOCE. However, the detailed explanation of the differentiation into a variety of phenotypes by the *STIM1* and *ORAI1* mutations is unclear. The cause of the phenotypical differences between TAM alone and Stormorken syndrome, and the distribution of involved muscles in TAM remains unknown. Recently-found molecules modulating STIM1 and ORAI1 interactions, such as Septin [95], SOCE-associated regulatory factor (SARAF) [96], CRAC regulator 2A (CRACR2A) [97], and STIM-activating enhancer (STIMATE) [98], increase the complexity, and their effects on the phenotype have not been well investigated. Therefore, a better understanding of the pathophysiology of Ca^2+^ signaling in addition to the components of SOCE and CRAC channels and development of therapeutic applications for TAM genes are expected.
